# Oral Administration of Bovine Milk from Cows Hyperimmunized with Intestinal Bacterin Stimulates Lamina Propria T Lymphocytes to Produce Th1-Biased Cytokines in Mice

**DOI:** 10.3390/ijms15045458

**Published:** 2014-03-28

**Authors:** Yuanyuan Wang, Lianjie Lin, Chunming Yin, Satoru Othtani, Katsuhiko Aoyama, Changlong Lu, Xun Sun, Yasunobu Yoshikai

**Affiliations:** 1 Department of Anesthesiology, the Fourth Affiliated Hospital of China Medical University, 4 Chongshan East Road, Huanggu District, Shenyang 110032, China; E-Mail: chunmingyin@hotmail.com; 2 Department of Digestive Medicine, Sheng Jing Hospital of China Medical University, No. 39 Huaxiang Road, Tiexi District, Shenyang 100022, China; E-Mail: audreylin73@hotmail.com; 3 Department of Immunology of China Medical University, 92 Bei’er Road, Heping District, Shenyang 110001, China; E-Mail: changlonglyu@hotmail.com; 4 Division of Host Defense, Medical Institute of Bioregulation, Kyushu University, 3-1-1 Maidashi, Higashi-ku, Fukuoka 812-8582, Japan; E-Mail: yoshikai@bioreg.kyushu-u.ac.jp; 5 Stolle Japan Corporation, 1-1-1 Shibuya, Tokyo 150-0002, Japan; E-Mail: otani@stolle.jp; 6 Orhto Corporation, 1-1-1 Shibuya, Tokyo 150-0002, Japan; E-Mail: aoyama@kenko.co.jp

**Keywords:** hyperimmune bovine milk, intestine, Th cell response, dextran sulphate sodium, colitis

## Abstract

The goal of this study was to examine the effects of oral administration of bovine milk from cows hyperimmunized with a proprietary bacterin (immune milk “Sustaina”) on mucosal immunity in the intestine of adult mice. C57BL/6 mice were orally given immune or control milk for two weeks, and then lymphocyte population and the cytokine production in lamina propria of colon in normal mice and mice induced colitis by dextran sulphate sodium (DSS) were detected. We found that the levels of IFN-γ and IL-10 increased, but the levels of IL-17A and IL-4, decreased in lamina propria of colon in immune milk-fed mice as compared with those in control milk-fed mice. Interestingly, oral administration of immune milk partially improved the acute colitis induced by DSS. The levels of TNF-α and IFN-γ increased, but IL-6, IL-17A and IL-4 decreased in lamina propria (LP) of colon in immune milk-fed mice with DSS-induced colitis. Our results suggest that immune milk may stimulate CD4^+^ T cells to polarize towards a Th1 type response, but contrarily suppress Th17 and Th2 cells responses in large intestinal LP of mice. The results indicate that this kind of immune milk has is able to promote the maintainance of intestinal homeostasis and enhance protection against infection, and could alleviate the symptoms of acute colitis in mice.

## 1. Introduction

The mucosa of the gastrointestinal tract is the first line of defense in battling invasion of non-self antigens from commensal microflora and foods, and constitutively active in terms of immunological aspect [1]. This unique environment utilizes an immune system mediated by Th1, Th2, Th17 and Treg cells that play important roles in homeostasis in the intestine through the production of various cytokines [2]. However, imbalance of these Th cell responses in intestine causes inflammatory bowel diseases (IBD) including Crohn’s disease (CD) and ulcerative colitis (UC) [3]. Among various experimentally induced colitis models in mice, dextran sulphate sodium (DSS)-induced colitis is one of the most popular murine colitis models. Although DSS-induced colitis is originally considered as a T cell-independent model [4], Th1-like responses are closely associated with pathogenesis, especially in the recovery phase of exaggerated colitis induced by DSS [5]. Furthermore, it has been recently reported that a novel subset of helper CD4^+^ T cells producing IL-17A, namely Th17 cells, is also involved in progression of DSS-induced colitis [6,7]. Also, regulatory CD4^+^ T cells, including naturally occurring Treg cells, inducible Treg cells, T helper type 3 (Th3) cells and T regulatory cell 1 phenotype (Tr1) cells play important roles in homeostasis in intestine through TGF-β and IL-10 production [8].

Recent advances in studies of gut microflora indicate that the composition of bacteria type such as *Clostridia* and *Lactobacilli* induce lamina propria (LP) Th polarization [9] and *Bifidobacteria* and *Lactobacilli* as probiotics [10] direct allergic Th2 responses in favour of Th1 responses. Colonization of segmental filamentous bacteria results in accumulation of Th17 cells [9], and cluster IV and XIVa *Clostridia* [11] and *Bacteriodes fragilis* [12,13] induce Treg responses in intestinal T cells. Thus, individual commensal species influence the composition of the lamina propria Th subsets that have distinct effector functions. In 1957, Stolle produced a kind of bovine milk that was termed “hyperimmunized milk” (immune milk) [14]. It was obtained from cows immunized with various human gut bacteria, including *Escherichia coli*, *Salmonella thyphimurium*, *Shigella dysenteriae*, *Staphylococcus pyogenes*, *Proteus vulgaris* and others. This kind of milk is characterized by higher amounts of immunoglobulins specific to these gut bacteria. As we previously reported, immune milk-administration protected mice from opportunistic infection induced by 5FU and irradiation [14,15]. It improved autoimmune diseases assessed by survival rate, proteinuria and anti-DNA Abs in autoimmune disease-prone mice (NZBxNZW F1 mice) [16] and it also delayed age-related impairment of T cell responses [17]. Our previous studies revealed the significant differences in the number and composition of gut microflora between immune milk-fed mice and control milk-fed mice [14–17]). These may stimulate the growth of beneficial microorganisms and influence the mucosal immunity in the intestine. However, it remains unknown whether immune milk may affect intestinal mucosal immunity and the development of intestinal inflammation.

In this study, in order to determine whether immune milk affects the activation of T cells in the lamina propria lymphocytes (T-LPL) of colon in mice, we examined the cytokine production by T-LPL of mice that were fed immune milk and the effect of immune milk-feeding on the development of DSS-induced colitis.

## 2. Results

### 2.1. Populations of LPL in Large Intestine of Mice Fed Immune Milk

**Figure 1.**
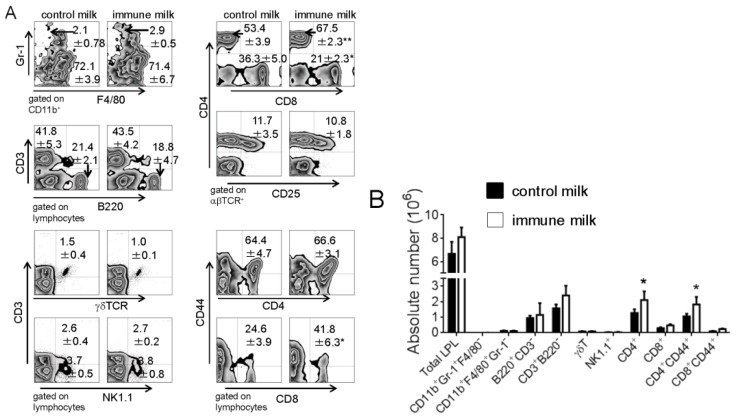
Flow cytometry analysis of the populations of LPL in the colon of mice before and after immune milk administration. (**A**) the frequency of neutrophil, macrophage, CD4^+^, CD8^+^, CD4^+^CD44^+^, CD8^+^CD44^+^, CD4^+^CD25^+^, γδ T cell, NK or NKT cell in LP of colon on day 14 after oral administration of immune milk; (**B**) the absolute cell number. Data indicate mean ± SD of 10 mice of each group obtained from a representative of three independent experiments (* *p* < 0.05; ** *p* < 0.01).

The subpopulations of LPL in mice were analyzed by flow cytometry two weeks after oral administration of immune milk. As shown in Figure 1A, the percentage of CD4^+^ T cells in T-LPL significantly increased, however the percentage of CD8^+^ T cells in T-LPL markedly decreased in large intestine of mice treated with immune milk as compared with those in control mice. The percentage of CD44^+^CD8^+^ cells in CD8^+^ T-LPL cells of mice after immune milk treatment was higher than that in control mice, but there was no difference in the percentage of CD44^+^CD4^+^ cells in CD4^+^ T cells between immune milk-fed mice and control milk-fed mice. The absolute numbers of CD4^+^ and CD4^+^CD44^+^ T-LPL cells significantly increased in mice after two weeks oral administration of immune milk when compared with those in control mice (Figure 1B). There were no significant differences in the frequencies and the absolute numbers of neutrophil (CD11b^+^Gr-1^+^F4/80^−^), macrophage (CD11b^+^Gr-1^−^F4/80^+^), NK cell (NK1.1^+^CD3^−^), NKT cell (NK1.1^+^CD3^+^), T cell (CD3^+^B220^−^), CD4^+^CD25^+^ T cell, γδ T cell and B cell (CD3^−^B220^+^) between immune milk-fed mice and control milk-fed mice. These results suggest that immune milk feeding may promote the proliferation of CD4^+^ T-LPL cells of colon in normal mice.

### 2.2. Cytokine Production by T-LPL Cells in Mice Fed Immune Milk

**Figure 2.**
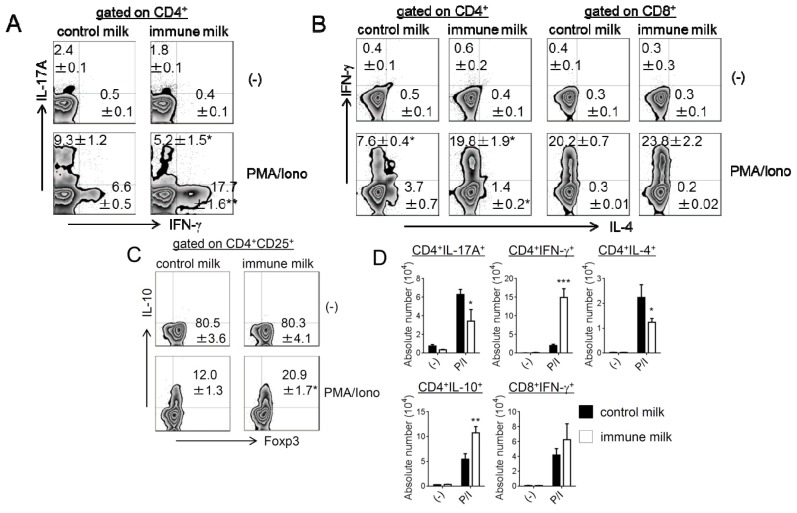
Cytokine-producing T cells in the LP of colon in mice before and after immune milk administration. (**A**) CD4^+^IFN-γ^+^ or CD4^+^IL-17A^+^ T-LPL; (**B**) IFN-γ- or IL-4-producing CD4^+^ or CD8^+^ T-LPL; (**C**) CD4^+^CD25^+^Foxp3^+^IL-10^+^ TLPL; (**D**) The absolute number of cytokine producing CD4^+^ or CD8^+^ T-LPL. Data indicate mean ± SD of 4 mice of each group obtained from a representative of three independent experiments (* *p* < 0.05; ** *p* < 0.01; *** *p* < 0.001).

**Figure 3.**
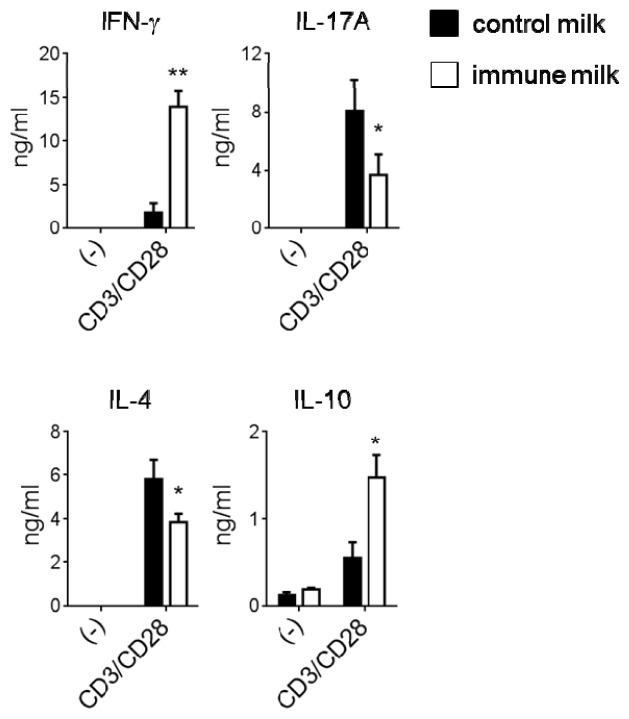
ELISA analysis of cytokine production in LP of colon in mice before and after immune milk administration. The level of IFN-γ, IL-17A, IL-4 or IL-10 production in the culture supernatants of LPL of colon was analyzed by ELISA before and after stimulation with anti-CD28/anti-CD3 mAbs. Data indicate mean ± SD of five mice of each group obtained from a representative of three independent experiments (* *p* < 0.05; ** *p* < 0.01). P/I: stimulation with PMA and ionomycin.

The frequency and number of IFN-γ, IL-17A, IL-4 or IL-10-producing CD4^+^ or CD8^+^ T-LPL were measured by intracellular cytokine staining. The numbers and frequencies of IFN-γ- and IL-10-producing CD4^+^ T-LPL were significantly higher in mice given immune milk, whereas IL-17A- and IL-4-producing CD4^+^ T-LPL were markedly lower in mice given immune milk than those in control mice (Figure 2). We also found that IL-10^+^ CD4^+^ T-LPL mainly expressed Foxp3, although not all Treg express Foxp3 (Figure 2C). However, there is no difference in Foxp3 expression on total CD4^+^ T-LPL before phorbol 12-myristate 13-acetate (PMA)/ionomycin stimulations between immune milk mice and control mice (Figure 2C). There is no difference in the frequency of IFN-γ-producing CD8^+^ T-LPL between immune milk-fed mice and control milk-fed mice (Figure 2B,D). We next measured the level of cytokine secretion by T-LPL in the large intestine of mice under the stimulations with immobilized anti-CD3 (10 μg/mL) and soluble anti-CD28 (1 μg/mL) mAbs. As shown in the Figure 3, IFN-γ and IL-10 secretions by T-LPL were significantly higher, but the levels of IL-17A and IL-4 were significantly lower in mice given immune milk than those in control mice. These results indicate that feeding immune milk may polarize Th1 response, and simultaneously suppress Th17 and Th2 responses in large intestinal LP of mice.

### 2.3. Immune Milk-Fed Mice Are not Susceptible to Induce Acute Colitis by DSS

**Figure 4.**
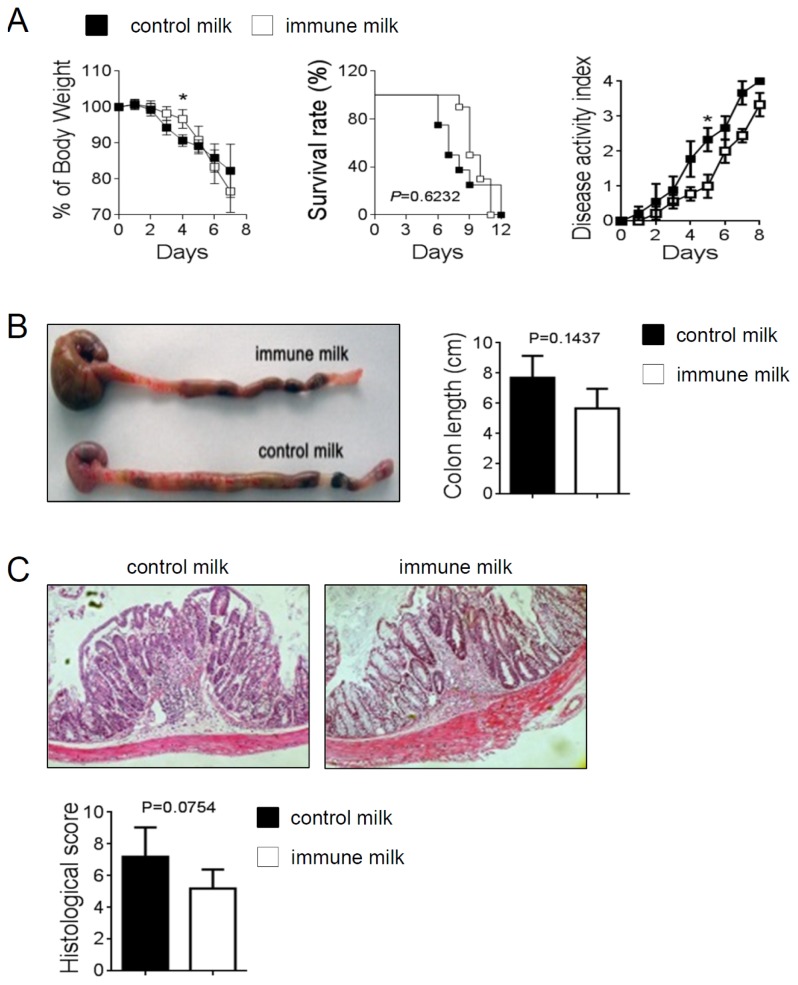
Symptoms of DSS-induced colitis in mice given immune milk were partially improved. (**A**) weight loss, survival rate, disease activity index; (**B**) macroscopic changes and colon length; (**C**) histological score (original magnification, ×200). Data indicate mean ± SD of 10 mice of each group obtained from a representative of three independent experiments (* *p* < 0.05).

Th cells including Th1, Th2 and Th17 cells, are known to be involved in the pathogenesis of DSS-induced colitis [17,18]. Therefore, we determined the influence of immune milk-feeding on the extent of DSS-induced acute intestinal inflammation in mice. Colitis was induced by oral administration of 3% DSS drinking water. Mice given immune milk showed a relatively minor intestinal inflammation to mice given control milk, as the weight loss and disease activity index significantly were improved on day 4 and day 5 respectively (Figure 4A). However, there was no difference in survival rate, colon length or histological score between immune milk-fed mice and control mice (Figure 4B,C). These results demonstrate that this kind of immune milk-feeding can partially alleviate the symptoms of acute colitis in mice.

**Figure 5.**
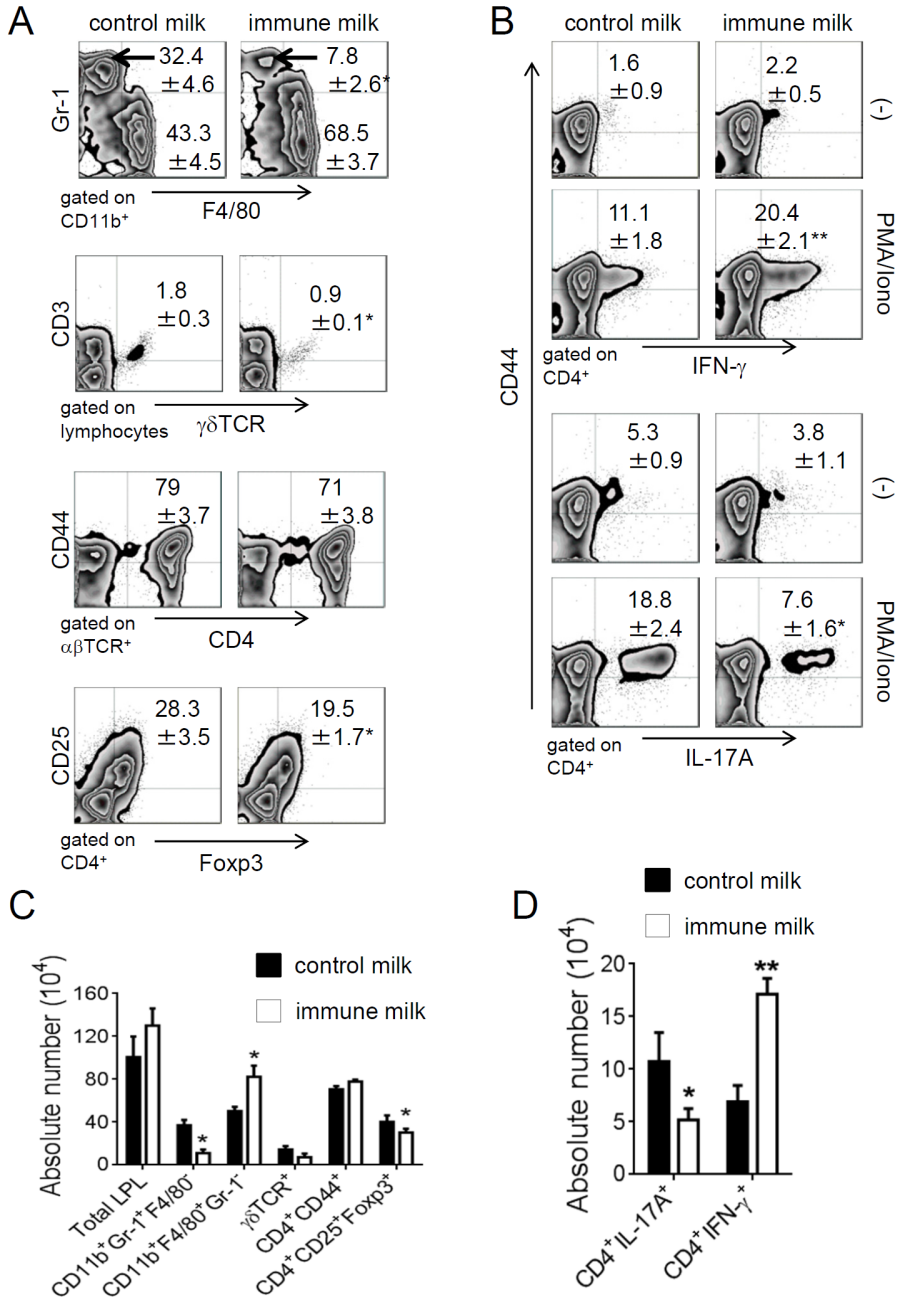
The populations of LPL and the cytokines producing T-LPL in colon of mice give immune milk after DSS-induced colitis were analyzed by Flow cytometry. (**A**) and (**B**) the frequencies of neutrophil, macrophage, CD4^+^CD44^+^, CD4^+^CD25^+^Foxp3^+^, γδ T cell, and IFN-γ- or IL-17A-producing CD4^+^CD44^+^ T-LPL were analyzed by flow cytometry on day 5 after administration with DSS; (**C**) and (**D**) the absolute cell numbers were counted. Data indicate mean ± SD of 5 mice of each group obtained from a representative of three independent experiments (* *p* < 0.05; ** *p* < 0.01).

Next, the population of LP cells in colon was analyzed by flow cytometry. The percentage and the number of Gr-1^+^ neutrophil significantly were decreased, while the number of F4/80^+^ macrophage was increased in immune milk-fed mice as compared with those in control mice on day 5 after induction of colitis. The percentage and the number of CD4^+^CD25^+^Foxp3^+^ T cells were decreased in LP of immune milk-fed mice (Figure 5A,C). The frequency of γδ T cells was also deceased in immune milk-fed mice (Figure 5A). On the other hand, the frequency and the number of IFN-γ-producing CD4^+^CD44^+^ T-LPL cells were significantly higher, but IL-17A-producing CD4^+^CD44^+^ T-LPL cells were significantly lower in immune milk-fed mice than those in control mice on day 5 (Figure 5B,D).

**Figure 6.**
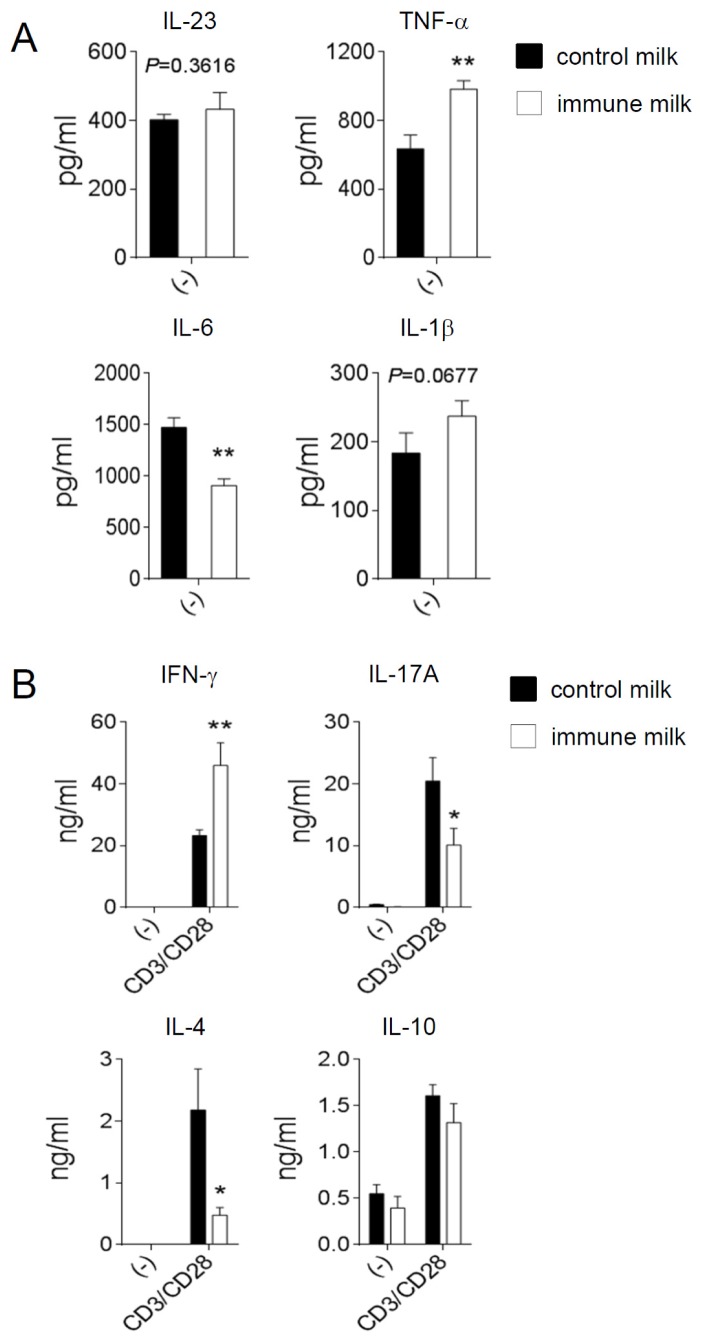
Analysis of cytokine secretions in large intestinal LP of immune milk fed mice after DSS-induced colitis. LPLs were isolated from the large intestine of mice on day 5 after DSS administration, and were cultured for 24 h without any stimulation (**A**) or were cultured for 48 h with TCR stimulations (anti-CD3/anti CD28 mAbs); (**B**) cytokine secretions in the culture supernatants were then measured by ELISA. Data indicate mean ± SD of 5 mice of each group obtained from a representative of three independent experiments (* *p* < 0.05; ** *p* < 0.01).

We also measured the level of cytokine in LP of large intestine. The level of inflammatory cytokine, TNF-α was higher, but IL-6 was lower in immune milk-fed mice than those in control mice on day 5 after induction of colitis (Figure 6A), whereas there was no difference in the level of IL-1β or IL-23 between immune milk-fed mice and control mice. The level of IFN-γ was significantly higher, however the levels of IL-17A and IL-10 were lower in immune milk-treated mice than those in control mice (Figure 6B). Therefore, these results suggest that immune milk feeding can regulate Th1, Th17 and Th2 cell responses in the intestinal LP of mice and partially alleviate the symptoms of DSS-induced acute colitis.

## 3. Discussion

We show here that oral administration of immune milk causes Th1-biased responses by T cells in LP of intestine of mice. CD4^+^ T-LPL cells from immune milk-treated mice showed Th1-like responses characterized by intact IFN-γ upon TCR triggering. However, other Th cell responses including Th17 and Th2 were downregulated in LP of immune milk-fed mice. It has been widely accepted that intestinal microflora plays important roles not only in inhibiting colonization by many pathogens and stimulating the growth of beneficial microorganisms but also in priming mucosal immunity. It could be of clinical interest to manipulate colonic flora because it is supposed that specific bacteria in the gut microbial microflora could promote potentially anti-microbial and anti-allergenic processes. Recent advance of studies of gut microflora indicate that the composition of bacteria type such as *Clostridia* and lactobacilli induce lamina propria Th polarization [1,13]. *Bifidobacteria* and *Lactobacilli* [9] direct allergic Th2 responses in favour of Th1 responses and colonization of segmental filamentous bacteria (SEB) results in accumulation of Th17 cells and cluster IV and XIVa *Clostridia* and *Bacteriodes fragilis* induced Treg responses in intestinal T-LPL cells [10–12]. Thus, individual commensal species influence the composition of lamina propria Th subsets, which have distinct effector functions. Immune milk contains abundant levels of Abs against various human gut bacteria, which cause human diseases associated with autoimmune disorders. Our previous studies revealed that there were significant differences in the number and composition of gut microflora between immune milk-fed mice and control milk-fed mice [14–16,19]. The number of enterobacteria significantly decreased in mice given immune milk only for 7 days, suggesting that immune milk can neutralize some of mouse gut bacteria. It may stimulate the growth of beneficial microorganisms such as *Bifidobacteria* and *Lactobacilli* and redirect allergic Th2 responses in favour of Th1 response. These results suggest a change in the microflora composition in adults by even short-term oral administration of immune milk shifts Th balance to Th1 response. Further analysis of microbiota using next-generation sequencing technologies is necessary to clarify this possibility.

We previously reported that immune milk could protect mice from indigenous infection induced by 5FU or irradiation, which damage intestinal epithelium and bone marrow, resulting in increased permeabilty and impaired host defense mechanism [15]. Immune milk may directly inhibit the invading pathogens in indigenous infection but also enhance local Th1 response which is most important for host defense against bacterial infection. We further reported that immune milk prevented Th2-related autoimmune diseases in autoimmune disease-prone mice (NZBxNZW F1 mice) and age-related decline of Th1 responses [16]. These may be explained by polarizing Th1 response in intestine by immune milk-feeding. Th1-like response is closely associated with pathogenesis, especially in the recovery phase of exaggerated colitis induced by DSS [18]. Furthermore, it has been recently reported that a novel subset of helper CD4^+^ T cells producing IL-17A, namely Th17 cells, is involved in progression of DSS-induced colitis [17], although an IL-17-mediated protective effect on the Th1-type colitis model has recently been reported [7]. In the present study we found that DSS-induced colitis was not exacerbated in immune milk-fed mice, although IFN-γ production increased in LP, but IL-17A and IL-4 productions decreased. Th1 cells inhibit the proliferation of Th2 cells, and Th2 cells shut down IFN-γ production by Th1 cells [1], indicating that Th1 and Th2 cells are mutually regulated. Th1 and Th17 cells are also known to be mutually regulated by induction of SOCS and STAT3 [20]. In the present study, Th1 responses characterized by IFN-γ production was augmented, but Th2 and Th17 responses were impaired after induction of colitis by DSS. Therefore, it is possible that Th1biased response by immune milk may regulate pathologic T cell responses in intestine after induction of DSS-induced colitis. Unique subpopulations of a third type of Th cells termed Th3 cells, Tr1 cells or Treg cells have recently been reported to play important roles in immune regulation through TGF-β and/or IL-10 production [8]. Therefore, it is also possible that Treg cells producing IL-10 may be activated by oral administration of immune milk. However, in the present study, IL-10-production by T-LPL was rather impaired in immune milk-fed mice after induction of colitis by DSS, excluding this possibility.

## 4. Materials and Methods

### 4.1. Milk Samples

Two kinds of skim milk were kindly provided by the Stolle Japan Corpration (Tokyo, Japan). Immune milk was obtained from cows immunized with a variety of human gut bacteria listed in Table 1. The cows were immunized intramuscularly with the bacteria mixture four times every week before delivery and twice every fortnight after delivery. Milk samples were obtained 2 weeks after the last vaccination. Control milk was obtained from unimmunized cows. The concentration of IgG1 in immune milk was by 20%–40% higher than control milk, and immune milk had significantly higher antibody activities against these bacteria. The nutritional composition was not significantly different between immune milk and control milk [19].

### 4.2. Mice

Sex and age-matched C57BL/6 and BALB/c mice were purchased from Charles River Japan (Yokohama, Japan). All mice were maintained under specific pathogen-free conditions and were offered food and water *ad libitum* and were used at 6–8 weeks of age. This study was approved by the Committee of Ethics on Animal Experiment in Faculty of Medicine, Kyushu University. Experiments were carried out under the control of the Guidelines for Animal Experiments. Mice were maintained under specific pathogen-free conditions and were fed with a solid diet including immune milk or control milk at 150 g·kg^−1^·day^−1^.

### 4.3. Abs and Reagents

Abs for flow cytometry analysis, Fcγ receptor-blocking mAb (CD16/32; 2.4G2), anti-CD3ɛ (145-2C11), anti-Gr-1 (RB6-8C5), anti-CD4 (RM4-5), anti-CD62L (MEL-14), anti-CD44 (IM7), anti-IL-17A (TC11-18H10.1), anti-IL-10 (JES5-16E3) and anti-IFN-γ (XMG1.2) were purchased from BD Biosciences (San Diego, CA, USA). Purified anti-CD3 and anti-CD28 (37.51) were obtained from e-Bioscience (San Diego, CA, USA). anti-F4/80 (BM8), anti-B220 (RA3-6B2), anti-TCRβ (H57-597), anti-CD8 (53–6.7), anti-NK1.1 (PK136), anti-TCRγδ (GL3), anti-CD25 (PC61), anti-IL-4 (11B11), anti-Foxp3 (MF-14) were purchased from Biolegend (San Diego, CA, USA).

### 4.4. Cell Preparation

LP cells in the colon were isolated by a modified method described previously [18]. In brief, gut pieces were cut into 2-mm samples and the epithelium was eliminated by stirring, first twice for 10 min in PBS containing 3 mM EDTA at 37 °C and then twice for 15 min in RPMI (Sigma Chemical Co., St. Louis, MO, USA) containing 1% FBS, 1 mM EGTA, and 1.5 mM MgCl2. Gut pieces were collected and stirred at 37 °C for 90 min in RPMI containing 20% FBS, 100 U/mL collagenase (C2139; Sigma-Aldrich Corp., St. Louis, MO, USA), and 5 U/mL DNase 1 (Sigma-Aldrich Corp.). Halfway through the incubation and at the end of the incubation, the suspension was dissociated by multiple aspirations through a syringe for 2 min. The pellet was washed and in some experiments LP cells were purified to LP lymphocytes (LPLs) on a 45%/66.6% discontinuous Percoll^®^ (Pharmacia, Uppsala, Sweden) gradient at 600× *g* for 20 min. The number of viable cells was counted by trypan blue or tulk’s solution staining.

### 4.5. Flow Cytometry

Isolated LPL cells from large intestine of mice were incubated with an FcγR-blocking mAb, and stained with mAbs against mouse F4/80, Gr-1, CD3, B220, NK1.1, TCRγδ, CD4, CD8, CD25 or CD44. For intracellular cytokine staining, LPL were stimulated with PMA, (25 ng/mL; Sigma-Aldrich) and ionomycin (1 μg/mL, Sigma-Aldrich) for 5 h at 37 °C. Brefeldin A (BFA, 10 μg/mL; Sigma-Aldrich) was added for the last 4 h of incubation. These cells were harvested, washed and stained with mAbs against mouse Foxp3, IL-10, IL-4, IL-17A or IFN-γ for 30 min at 4 °C. The intracellular expression of cytokine in CD4^+^ or CD8^+^ T-LPL was analyzed by intracellular cytokine FACS, using a Cytofix/Cytoperm Kit Plus (BD Biosciences, San Diego, CA, USA) according to the manufacturer’s instructions. The data were analyzed using CellQuest software (BD Biosciences).

### 4.6. Cytokine Enzyme-Linked Immunosorbent Assay (ELISA)

To measure spontaneous cytokine production by LPL of colon, 2 × 10^5^ LPL were cultured without any stimulation for 24 h at 37 °C under 5% CO_2_ in 96-well flat-bottomed plates in a volume of 0.2 mL RPMI 1640 (Wako, Japan) containing 10% fetal bovine serum (FBS) (Cell Culture Technologies, Tokyo, Japan). To measure cytokine production by T-LPL cells, 2 × 10^5^ LPL were cultured with 96-well flat-bottom plates (Falcon; BD Biosciences) coated with anti-CD3 (10 μg/mL) and soluble anti-CD28 mAbs (1 μg/mL) for 48 h. IL-23, TNF-α, IL-6, IL-1β, IFN-γ, IL-17A, IL-4 or IL-10 secretion in the culture supernatants was measured by using ELISA Kit (Genzyme Diagnostics, Cambridge, MA, USA) according to the manufacturer’s protocols.

### 4.7. Induction of Acute Colitis by DSS

For acute colitis induction by DSS, mice were administered 3% (weight/volume) DSS (molecular weight 36–50 kilodaltons; ICN Biomedicals, Aurora, OH, USA) in their drinking water. Mice were sacrificed on day 5 in acute colitis. The tissues of colons were removed and cleaned. Sections were taken for cell culture, flow cytometry or histological analysis.

### 4.8. Histology Assessment of Colitis

The middle parts of colons were removed and fixed with 10% neutral buffered formalin and then embedded in paraffin. Five mm tissue sections were stained with hematoxylin and eosin (HE). Histology was scored as follows: epithelium (E), 0 = normal morphology; 1 = loss of globlet cells; 2 = loss of globlet cells in large areas; 3 = loss of crypts; 4 = loss of crypts in large areas; and infiltration (I), 0 = no infiltrate; 1 = infiltrate around the crypt basis; 2 = infiltrate reaching the L. muscularis mucosae; 3 = extensive infiltration reaching the L. muscularis mucosae and thickening of the mucosa with abundant edema; 4 = infiltration of the L. submucosa. The total histological score was given as E + I. [21].

### 4.9. Statistical Analysis

The difference in survival rates was evaluated by the log rank test (Mantel-Cox). Differences in parametric data were evaluated by a Student’s *t* test. Differences of *p <* 0.05 were considered statistically significant.

## 5. Conclusions

Oral administration of immune milk caused Th1-like response by T-LPL but not aggravated DSS-induced colitis. Moreover, immune milk-treatment may partly alleviate the symptoms of colitis such as UC by downregulation of Th17 and Th2 responses in gut. Immune milk-feeding also may be useful to reduce allergic disease and to prevent infectious diseases and malignancy without aggravating IBD. There is thus much interest in determining the protective efficacy of oral supplementation with immune milk.

## Figures and Tables

**Table 1.** Composition of immune milk and control milk. Bacterial antigens used for bovine immunization and antibody activity of milk samples 1.

Bacterial antigen	ATCC No. 2	IgG 3 (μg/g milk)	

		Control milk	Immune milk
*Staphylococcus simulans*	11631	10.8	NT
*Staphylococcus epidermidis*	155	NT	15.2
*Staphylococcus pyogenes*, Type 1	8671	NT	13.6
*Staphylococcus pyogenes*, Type 3	10389	1.9	90.6
*Staphylococcus pyogenes*, Type 5	12347	2.2	24.4
*Staphylococcus pyogenes*, Type 8	12349	11.4	34
*Staphylococcus pyogenes*, Type 12	11434	2.5	77
*Staphylococcus pyogenes*, Type 14	12972	5.3	80.0
*Staphylococcus pyogenes*, Type 18	12357	10.7	88.4
*Staphylococcus pyogenes*, Type 22	10403	12.4	106.2
*Aerobacter aerogenes*	884	14.2	52.5
*Escherichia coli*	26	16.0	109.0
*Salmonella enteritidis*	13076	12.5	158.3
*Pseudomonas aeruginosa*	7700	18.7	68.4
*Klebsiella pneumoniae*	9590	42.9	305.0
*Salmonella typhimurium*	13311	21.5	46.2
*Haemophilus influenzae*	9333	36.3	50.9
*Streptococcus mitis*	6249	9.8	36.4
*Proteus vulgaris*	13315	16.2	28.1
*Sigella dysenteriae*	11835	12.5	43.4
*Propionibacterium acnes*	11827	12.6	20.9
*Streptococcus sanguis*	10556	12.5	44.0
*Streptococcus salivarius*	13419	6.8	30.6
*Streptococcus mutans*	25175	9.1	31.0
*Streptococcus agalactiae*	13813	6.2	30.6
*Streptococcus pneumoniae*	6303	6.2	31.0

1 Antibody concentration was measured using the dried sterilized milk by enzyme immunoassay;

2 American Type Culture Collection (1991), Rockville, MD, USA;

3 Values are means, *n* = 3;

NT: not tested.
